# Internalization of silver nanoparticles into mouse spermatozoa results in poor fertilization and compromised embryo development

**DOI:** 10.1038/srep11170

**Published:** 2015-06-08

**Authors:** Ton Yoisungnern, Yun-Jung Choi, Jae Woong Han, Min-Hee Kang, Joydeep Das, Sangiliyandi Gurunathan, Deug-Nam Kwon, Ssang-Goo Cho, Chankyu Park, Won Kyung Chang, Byung-Soo Chang, Rangsun Parnpai, Jin-Hoi Kim

**Affiliations:** 1 Department of Animal Biotechnology, College of Animal Bioscience and Biotechnology/Animal Resources Research Center, Konkuk University, Seoul 143-701, South Korea; 2 Embryo Technology and Stem Cell Research Center, School of Biotechnology, Suranaree University of Technology, Nakhon Ratchasima 30000, Thailand; 3 Department of Cosmetology, Hanseo University, Seosan, Chungnam 356-706, Korea

## Abstract

Silver nanoparticles (AgNPs) have many features that make them attractive as medical devices, especially in therapeutic agents and drug delivery systems. Here we have introduced AgNPs into mouse spermatozoa and then determined the cytotoxic effects of AgNPs on sperm function and subsequent embryo development. Scanning electron microscopy and transmission electron microscopy analyses showed that AgNPs could be internalized into sperm cells. Furthermore, exposure to AgNPs inhibited sperm viability and the acrosome reaction in a dose-dependent manner, whereas sperm mitochondrial copy numbers, morphological abnormalities, and mortality due to reactive oxygen species were significantly increased. Likewise, sperm abnormalities due to AgNPs internalization significantly decreased the rate of oocyte fertilization and blastocyst formation. Blastocysts obtained from AgNPs-treated spermatozoa showed lower expression of trophectoderm-associated and pluripotent marker genes. Overall, we propose that AgNPs internalization into spermatozoa may alter sperm physiology, leading to poor fertilization and embryonic development. Such AgNPs-induced reprotoxicity may be a valuable tool as models for testing the safety and applicability of medical devices using AgNPs.

The application of nanoparticles (NPs) is widespread and has been extensively used in therapeutic and diagnostic agents, drug delivery systems, medical devices, food containers, and cosmetics^1^,^2^,^3^. Silver nanoparticles (AgNPs) are among the most popular nanomaterials used in material science, most importantly as the constituents of dental alloys, catheters, and implant surfaces; for treating wound and burn-related infections; and in drug delivery in cancer and retinal therapies^4^,^5^,^6^. Therefore, both consumers and the workers manufacturing these products are exposed to AgNPs, which may have harmful effects.

Several studies have demonstrated the effects of subchronic oral or inhalation toxicity of AgNPs in experimental animals. They also found that silver was accumulated in the blood and all tested organs, including the liver, spleen, kidneys, thymus, lungs, heart, brain, and testes^6^,^7^. The mechanism by which NPs can induce cytotoxicity is thought to be by increasing intracellular oxidative stress and apoptosis^8^,^9^,^10^,^11^,^12^,^13^. Like other nanoparticles, AgNPs also show risk of toxicity by generating reactive oxygen species (ROS)^14^,^15^. Several studies suggest that the toxicity of AgNPs is mainly mediated by the release of silver ions (Ag^+^)^16^. AgNPs can enter the cell by diffusion or endocytosis to cause mitochondrial dysfunction, leading to damage of proteins and nucleic acids, ultimately inhibiting cell proliferation^17^,^18^,^19^,^20^.

The influence of NPs on a single gamete may cause remarkable developmental differences as gamete quality plays a crucial role in gametogenesis^21^. Impairment of gametes due to exposure to NPs may affect reproductive functions or have pathological influences on the next generation^22^. However, studies on the sensitivity of gametes to NPs exposure are very limited. In spermatozoa, polyvinyl alcohol- and polyvinyl pyrrolidone (PVP)-coated iron and europium hydroxide NPs do not show any toxicity^23^. Titanium dioxide, gold, silver, and zinc oxide NPs show moderate effects^24^,^25^,^26^,^27^,^28^. On the other hand, europium trioxide shows severe cytotoxicity in spermatozoa^29^.

A literature survey shows only a few studies on the effects of AgNPs on fertility and sperm function. AgNPs exposure has been shown to affect testicular morphology, reduce sperm production, and increase the number of abnormal spermatozoa and germ cell DNA damage *in vivo*^30^,^31^,^32^,^33^. In another *in vivo* study in rats, Miresmaeili *et al.*^34^ showed that AgNPs exposure significantly decreased the number of spermatogenic cells, including spermatogonia, spermatocytes, spermatids, and spermatozoa, and also affected the acrosome reaction in sperm cells. Several *in vitro* studies also showed that AgNPs caused cytotoxicity/apoptosis in testicular cells and embryos, and affected the proliferation rate in spermatogonial stem cells^35^,^36^,^37^,^38^. In another *in vitro* study, *Moretti et al.*^26^ showed that AgNPs exerted a significant dose-dependent effect on motility and viability of human spermatozoa. However, extensive *in vitro* studies related to the effects of AgNPs on sperm parameters and the fertilization capacity of sperm during *in vitro* fertilization (IVF), as well as the effects on subsequent embryonic development are limited or not yet studied. More specifically, the mechanisms of AgNPs trafficking and uptake, compensating mechanisms of the surrounding tissues, or other potential confounders might explain the differences between *in vivo* and *in vitro* data.

So far, researchers have focused on the binding and internalization of AgNPs into sperm cells and its dose-dependent cytotoxic effects in spermatozoa before IVF. Our study is the first to report the effects of AgNPs-treated sperm on subsequent IVF- or intracytoplasmic sperm injection (ICSI)-derived embryonic development. Therefore, the aims of our present study were to (i) identify the cytotoxic effect of AgNPs on spermatozoa, (ii) evaluate the effect of AgNPs on sperm acrosome reaction, (iii) assess the effect of AgNPs on sperm fertilization capacity during IVF and embryonic development, (iv) understand the role of AgNPs on cell proliferation in blastocysts, and (v) explore the effect of AgNPs on inner cell mass (ICM)- and trophectoderm cell (TE)-specific genes expression in blastocysts.

## Results

### Characterization of AgNPs

The diameter and morphology of AgNPs, shown in Supplementary Figs. 1a and 1b, were analyzed by transmission electron microscopy (TEM). The representative TEM image indicated well-dispersed particles that were more or less spherical. We measured the diameter of more than 300 particles and the distribution is represented in Supplementary Fig. 1b. Although the average size was 40 nm, the AgNPs colloidal suspension contained different sized particles having diameter range mostly between 34 nm to 46 nm. Therefore, we actually used the AgNPs having particles diameter ranging from 34 nm to 46 nm (with average diameter of 40 nm) in our present study. We also measured the diameter of AgNPs by dynamic light scattering (DLS) and the distribution is depicted in Supplementary Fig. 1c. It was found that the average diameter of AgNPs analyzed by DLS was approximately 61.51 nm (Supplementary Fig. 1c). Further, AgNPs were characterized by ultraviolet (UV)-visible spectroscopy and X-ray diffraction (XRD). The UV-visible absorption spectra were measured in the range of 350–600 nm. The UV-visible spectra showed a strong and broad surface peak located at 420 nm (Supplementary Fig. 1d). The XRD pattern of AgNPs is shown in Supplementary Fig. 1e. We obtained five peaks at 2θ values of 38.2, 44.4, 64.5, 77.3 and 81.5° corresponded to Bragg's reflections from the (111), (200) (220), (311) and (222) planes respectively. The XRD results clearly showed that the AgNPs are crystalline in nature.

### Internalization of AgNPs in spermatozoa

**Figure 1:**
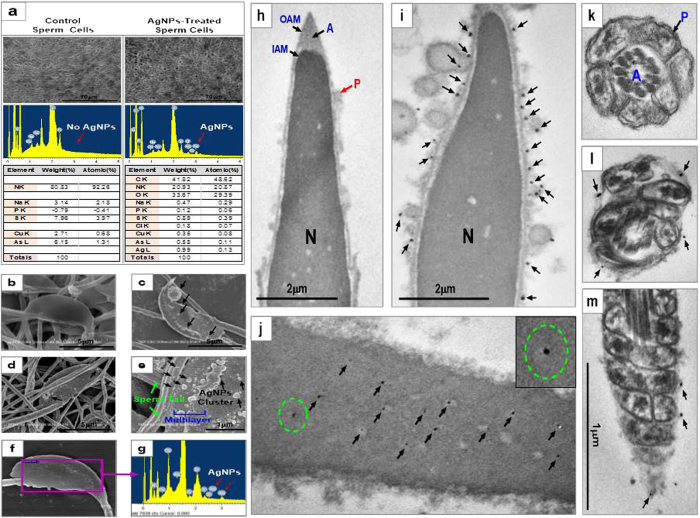
SEM and TEM analysis of AgNPs-treated sperms. SEM-EDS analysis: (**a**) AgNPs were detected in the AgNPs-treated group but not in the control. Energy dispersive spectroscopy (EDS) analysis showed an Ag peak only in AgNPs-treated group (red arrow) and contained 0.03% Ag among all atomic masses. (**b**) A representative SEM figure of a control sperm cell. (**c**~**e**) AgNPs detection in AgNPs-treated sperm cells: AgNPs cluster covered the head and tails of sperm cells. (**f** and **g**) Identification of AgNPs in sperm cells using EDS. Arrows indicated the AgNPs peak. (**h**) TEM analysis of a control sperm cell. (**i** and **j**) AgNPs were bound to the plasma membrane (**i**) or internalized into the head (**j**) of sperm cells. Open circle of (**j**) was magnified in the lower panel of (**j**). (**k**) Control mitochondria. P, cell membrane (**l** and **m**) Detection of AgNPs in mitochondria. Arrows indicate AgNPs. Note, due to internalization or binding of AgNPs, sperm cell mitochondria and axoneme were severely disorganized and/or distorted. P, A, N, OAM, and IAM indicated the plasma membrane, acrosome, nucleus, outer acrosome membrane, and inner acrosome membrane, respectively.

EDS profiling of sperm cells showed a weak signal for Ag along with weak signals of Cl, whereas we did not observe Ag in any of the control-treated spermatozoa (Fig 1a). On the basis of these findings, we examined AgNPs binding on spermatozoa using scanning electron microscopy (SEM). As shown in Fig. 1c–g, we found that AgNPs cluster on the midpiece or head of spermatozoa. SEM images of AgNPs indicated that they were more or less spherical in shape. Of note, EDS profiling of a sperm head showed a mild Ag signal (Fig. 1g), indicating that AgNPs were localized on the sperm head (Fig. 1f).

Next, we examined whether the 40 nm AgNPs had the ability to internalize into spermatozoa using TEM. Since AgNPs have high atomic numbers, it is possible to distinguish them from cellular structures using TEM. As the AgNPs dosage increased, internalization of AgNPs became more obvious inside the spermatozoa head from sperm plasma membrane and evenly dispersed in the sperm head and midpiece including the mitochondrial area (Figs. 1i,j,l). The degree of AgNPs internalization was clearly dose-dependent in the studied concentration range of 0.1 μg/mL to 50 μg/mL: at low dosages, AgNPs mainly localized on the sperm plasma membrane (Figs. 1i,k; Supplementary Fig. 2 and 3), whereas with increasing dosage, AgNPs were internalized into the head and mitochondrial area of the spermatozoa (Fig. 1j,l,m).

### Mitochondrial DNA copy number in AgNPs-treated spermatozoa

**Figure 2:**
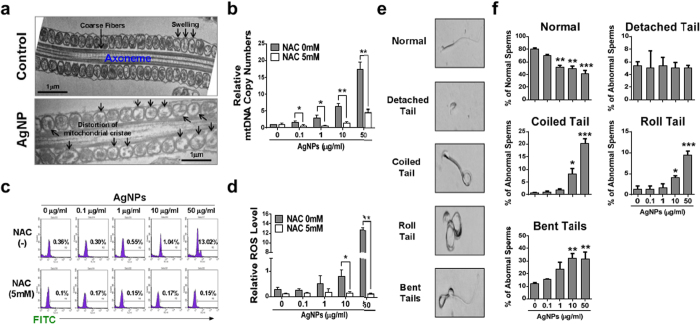
Mitochondrial damage, ROS analysis, abnormal sperm morphology analysis after treatment with different concentrations of AgNPs. (**a**) The mitochondrial sheath in control and AgNP-treated sperm cells was analyzed using TEM. Arrows indicate swelling of mitochondria in the mitochondrial sheath. (**b**) Determination of mitochondrial copy number in AgNPs-exposed sperms in presence or absence of NAC pre-treatment. The mtDNA/ACTB ratio, which represents the average copy number per sperm cell, was determined by qPCR. (**c** & **d**) ROS in NAC-pretreated or untreated sperm cells after exposure to AgNPs was analyzed using flow cytometry by staining with DCFH-DA-FITC. (**e**) A representative sperm morphological pattern, which was observed by phase contrast microscopy. (**f**) A minimum of 100 sperm cells per replicate with three replicates were investigated for morphology analysis and classified into five categories: normal morphology, detached head, coiled tail, roll tail, and bent tails. ^*^p < 0.05, ^**^p < 0.01, and ^***^p < 0.001 versus the control group (Dunnett’s *t*-tests).

After co-incubation with AgNPs for 3 h, the AgNPs-treated spermatozoa and control sperm cells were subjected to TEM for mitochondrial abnormality analysis and real-time quantitative polymerase chain reaction (qPCR) for determination of mitochondrial DNA (mtDNA) copy number. As shown in Fig. 2a (AgNPs-treated group, lower panel), most of the mitochondria derived from the AgNPs-treated spermatozoa were swollen, whereas control-derived spermatozoa were regular in shape. Axoneme and longitudinal fiber microtubules in different tail regions of many sperm cells were degenerated and had lost their architectural appearance. Completely distorted mitochondrial cristae were also observed in the midpieces of many sperm. Next, we examined mtDNA copy number. To estimate relative mtDNA copy number, we used real-time qPCR to amplify cytochrome b (Cytb) in mtDNA and actin beta (ACTB) in nuclear DNA. The mtDNA/ACTB ratio, which represents the average copy number per sperm cell, was determined in the 3 different samples. We found that AgNPs-treated sperm cells showed increased mtDNA copy number with increasing dose (correlation coefficient, r = 0.991) (Fig. 2b). In order to investigate the relationship between AgNPs-induced ROS formation and subsequent mitochondrial abnormalities in spermatozoa, the spermatozoa were pre-treated with *N*-acetyl-l-cysteine (NAC) followed by AgNPs intoxication, because NAC is a well known ROS scavenger. NAC pre-treatment for 30 min reduced the AgNPs-induced increased mtDNA copy number in spermatozoa. This observation suggested that mtDNA copy number appeared to be closely associated with AgNPs-induced oxidative stress.

### Reactive oxygen species analysis in AgNPs-treated spermatozoa

To examine the relationship between the increased mitochondrial damage induced by AgNPs and oxidative stress, reactive oxygen species (ROS) formation was quantified in spermatozoa exposed to AgNPs with or without NAC. As shown in Fig. 2c,d, flow cytometry analysis showed that AgNPs exposure significantly enhanced dichlorofluororescein (DCF)-positive signals in spermatozoa in a dose-dependent manner (correlation coefficient, r = 0.989). However, NAC pre-exposure reduced the percentage of DCF-positive sperm cells, indicating that ROS generation was reduced. These results were confirmed by fluorescence microscopy. Consistently, ROS generation induced by AgNPs results in high FITC fluorescence intensity (Supplementary Fig. 4), whereas pre-exposure to NAC inhibited AgNPs-induced ROS production.

### Effect of AgNPs on sperm morphology and sperm viability

**Figure 3:**
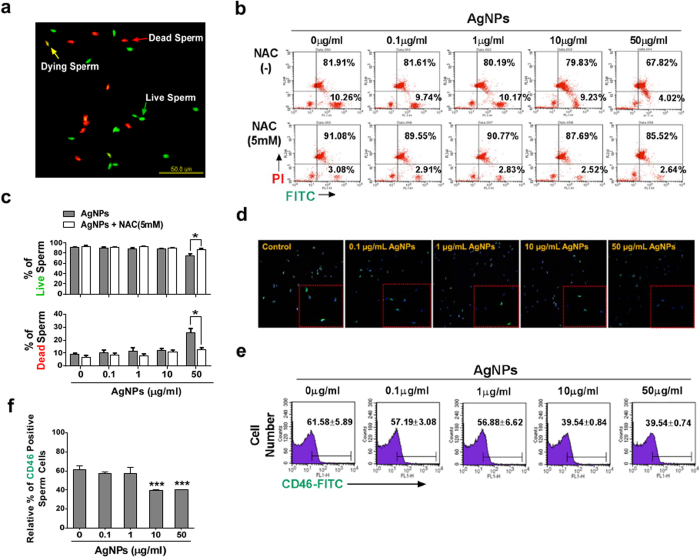
Sperm viability and acrosome reaction analysis. Sperm cells were treated with 0, 0.1, 1, 10, or 50 μg/mL AgNPs. For the ROS inhibitor group, sperm were pretreated with 5 mM NAC for 30 min, and then, 0–50 μg/mL of AgNPs was added. Dead and live sperm cells were counted using the Dead/Live kit. (**a**) Fluorescence microscopy image. Live sperms cells were stained with SYBR 14 dye (green fluorescence), whereas dead sperm cells were stained with propidium iodide (PI; red fluorescence). Yellow color in the merged image indicates dying sperm cells. (**b** & **c**) Dead and live sperm cells were calculated by flow cytometry: FL1 and FL2 represent green (live sperm) and red (dead sperm) color. (**d**) A representative immunofluorescence staining pattern obtained using mouse anti-CD46 (green) antibody. Nuclei are counterstained with DAPI (blue). (**e** & **f**) Flow cytometry analysis of CD46-positive sperm populations. Data for (**f**) was obtained from the flow cytometry experiment in (**e**). *p < 0.05, **p < 0.01, and ***p < 0.001 versus the control group (Dunnett’s *t*-tests).

As shown in Fig. 2e,f, sperm morphology was examined after incubation with different concentrations of AgNPs. In this study, there were 5 characteristics of spermatozoa: normal, detached head, coiled tails, roll tails, and bent tail (Fig. 2e). We found significant abnormal morphological changes in spermatozoa, such as coiled tail, roll tail, and bent tails at high concentrations of AgNPs (10 μg/mL and 50 μg/mL) (Fig. 2f). The major populations of live spermatozoa that stained with SYBR-14 (green color), dead spermatozoa that stained with propidium iodide (PI; red color), and merged spermatozoa that stained with both green and red (yellow color) are shown in Fig. 3a. Exposure of spermatozoa to AgNPs had a negative effect on sperm viability as measured by flow cytometry analysis. As shown in Fig. 3b, incubation of spermatozoa with AgNPs slightly increased the population of dead spermatozoa (correlation coefficient, r = 0.988). To confirm these results, sperm viability was checked by counting the number of dead and live spermatozoa, which were stained red and green, respectively. These results showed that only at the highest dose, AgNPs exposure significantly (*p* < 0.05) increased the percentage of dead spermatozoa, whereas the number of live spermatozoa was significantly decreased (Fig. 3c). Since silver ion (Ag^+^) exposure formed precipitation in the Modified Whitten’s medium, we could not examined whether Ag^+^ exposure significantly reduced sperm viability. Of note, pre-treatment with NAC for 30 min decreased the AgNPs-induced alterations in sperm viability (Fig. 3c). However, the viability of the spermatozoa cultured in non-capacitation (NCP) medium containing different AgNPs dosages (0.1, 1, 10, and 50 μg/mL AgNPs for 3 h) was more significantly decreased than those cultured in capacitation (CP) medium (Supplementary Fig. 5).

### Impact of AgNPs exposure on acrosome reaction

In this study, spermatozoa undergoing the acrosome reaction were evaluated with the help of CD46-positive signals using immunofluorescence staining (Fig. 3d). The population of CD46-positive spermatozoa was measured by flow cytometry analysis (Fig. 3e,f). AgNPs exposure affected the acrosome reaction of spermatozoa, causing significant (*p* < 0.001) reduction of the proportions of CD46-positive sperm in capacitation medium containing 10 (39.53% ± 0.84%) and 50 (40.08% ± 0.74%) μg/mL of AgNPs when compared to the control group (61.58% ±  5.89%) (Fig. 3d–f). To further confirm our results, spermatozoa were exposed to different concentrations of AgNPs in NCP medium. Our results suggested that AgNPs did not trigger the acrosome reaction (Supplementary Fig. 6 and 7), and we found that AgNPs exposure to spermatozoa reduced the acrosome reaction, as evident by decreased proportions of CD46-positive sperm.

### ***In vitro*** fertilization and ***In vitro*** culture assessment using living sperm cells after AgNPs exposure and subsequent blastocyst quality analysis

Spermatozoa derived from the ampulla of male BDF1 (8–12 weeks old) mice were randomly assigned to the control or AgNPs-treated groups. To examine the effect of AgNPs exposure on IVF and subsequent embryonic development, spermatozoa were treated with the presence or absence of various concentrations of AgNPs for three hours before IVF. We used an IVF system to evaluate the effect of AgNPs on oocyte fertilization and subsequent embryonic development. The percentage of unfertilized oocytes (44.4%, 65.2%, 77.0%) was significantly increased in spermatozoa treated with higher concentrations of AgNPs (1, 10, 50 μg/mL; *p* < 0.05) (Supplementary Table 1). In contrast, the rate of blastocyst formation was significantly decreased by the treatment of spermatozoa with AgNPs in a dose-dependent manner (*p* < 0.05).

**Figure 4:**
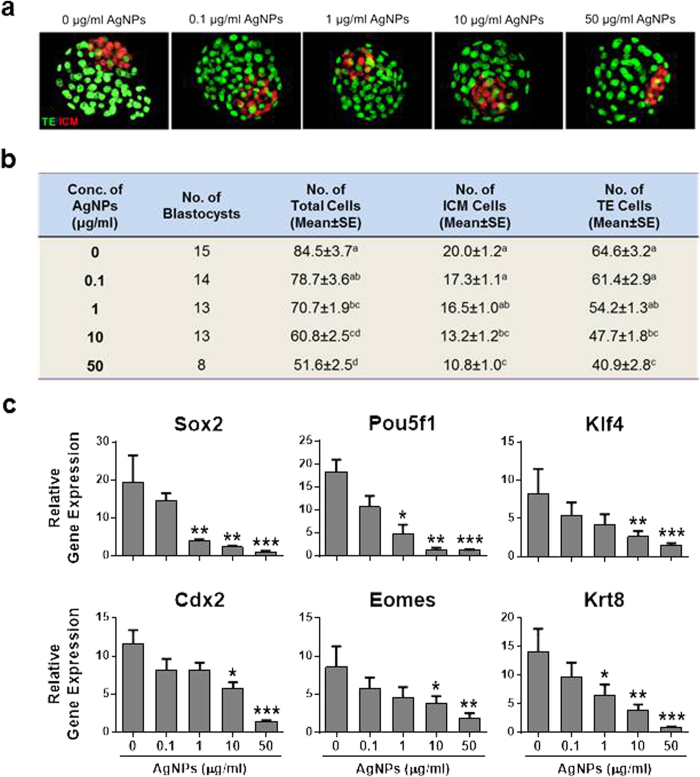
Qualitative analysis of blastocyst stage embryos developed *in vitro* after IVF with AgNP-treated sperm cells. (**a**) A representative blastocyst embryo was stained differentially by OCT4 and CDX2 antibodies 96 h after IVF with AgNP-treated sperm cells. OCT4-positive cells (red) are putative inner cell mass (ICM), whereas CDX2-positive cells (green) are putative trophectoderm. (**b**) Number of total cells, ICM, and TE cells from the blastocyst stage embryos of (**a**). (**c**) ICM- and TE-specific mRNA expression analysis in blastocysts developed after IVF with AgNPs-treated sperm cells. The expression levels of ICM and TE associated genes were analyzed by real time RT-qPCR. *p < 0.05, **p < 0.01, and ***p < 0.001 versus the control group (Dunnett’s *t*-tests).

To determine the effects of AgNPs-exposed spermatozoa on the quality of embryos, total number of cells, including the inner cell mass (ICM) and trophectoderm (TE) cells, in blastocysts at 96 h after *in vitro* culture (IVC) was determined by counting OCT4- and CDX2-positive signals, respectively (Fig. 4a). At 96 h after IVC, the total number of cells, ICM, and TE cells in blastocyst stage embryos derived from 1, 10, 50 μg/mL AgNPs-treated living spermatozoa were significantly decreased (*p* < 0.01) compared to control spermatozoa (Fig. 4b), suggesting that AgNPs exposure to sperm cells could reduce both ICM and TE cell numbers. Furthermore, pluripotent marker genes such as *SOX2*, *POU5F1*, and *KLF4* in blastocysts derived from AgNPs-exposed spermatozoa showed significantly lower expression with increasing AgNPs dosage. Besides, TE-associated genes such as *CDX2*, *EOMES*, and *KRT8* showed significantly lower expression in spermatozoa treated with high concentrations of AgNPs (10 and 50 μg/mL) compared to the control group (Fig. 4c).

### ICSI and IVC assessment using dead sperm after AgNPs exposure and blastocyst quality analysis

We next compared the oocyte fertilization and subsequent blastocyst development efficiency by injecting the sperm of first grade morphology (dead sperm with normal morphology) and second grade sperm (dead sperm with detached head or coiled tails) separately. Using ICSI, spermatozoa with different morphologies were selected after treatment with different concentrations of AgNPs. Of note, the percentage of fertilized oocytes and subsequent blastocyst development following ICSI were statistically different between first- and second-morphology grade spermatozoa (*p* < 0.05) (Supplementary Table 2). However, the rate of ICSI-derived oocyte fertilization and blastocyst formation using living sperm with normal morphology were significantly higher than those of AgNPs-treated dead sperm. Therefore, our study showed that sperm grading due to morphological difference was not correlated to ICSI outcome.

Next, we examined whether dead spermatozoa with abnormal morphology after AgNPs exposure would decrease embryo quality. Embryos were produced by ICSI using sperm cells with abnormal morphology after treatment with different dosages of AgNPs. At 96 hours post IVC, the total numbers of ICM and TE cells in blastocyst embryos were calculated by counting OCT4 and CDX2 positive signals, respectively (Supplementary Fig. 8a). These results showed that the dead spermatozoa with abnormal morphology (detached head, coiled tail) due to AgNPs exposure significantly (*p* < 0.01) reduced both ICM and TE cell numbers (Supplementary Fig. 8b). Of note, the dead spermatozoa with normal morphology from AgNPs treatment also showed a significant (*p* < 0.01) reduction in the number of ICM, TE, and total cells. Therefore, we concluded that AgNPs treatment decreased both ICM and TE cell numbers irrespective of sperm morphology.

To confirm the effect of AgNPs-exposed spermatozoa on ICM and TE-specific gene expression in ICSI-derived blastocyst embryos, embryos were produced by ICSI using dead spermatozoa with abnormal morphology due to AgNPs exposure. ICM and TE-specific gene expression in blastocyst embryos was studied by RT-qPCR as shown in Supplementary Fig. 8c. These results showed that ICM- and TE-specific gene expression in blastocyst embryos produced from dead spermatozoa with abnormal morphology due to AgNPs exposure was decreased relative to live spermatozoa-derived blastocyst stage embryos. However, the expression of ICM- and TE-specific genes in blastocysts derived from spermatozoa with abnormal morphology was not significantly different compared to dead sperm with normal morphology.

## Discussion

In this study, we examined the cytotoxic effect of AgNPs on spermatozoa, oocyte-fertilizing capacity of sperm, and subsequent embryonic development. The average diameter of AgNPs used in our study was found to be 40 nm and 61.51 nm as measured by TEM and DLS respectively. Further, AgNPs were characterized by UV-visible spectroscopy which showed a strong and broad surface peak located at 420 nm. The XRD results also clearly showed that the AgNPs are crystalline in nature.

Spermatozoa obtained from male BDF1 mice were treated with AgNPs at a dose of 0, 0.1, 1, 10, and 50 μg/mL for 3 h, and the treated spermatozoa were used for IVF or ICSI of oocytes followed by embryonic culture *in vitro* for 96 h. Our results showed that AgNPs could internalize into the spermatozoa. When the AgNPs were applied at a lower dosage, they mainly localized on the sperm plasma membrane. On the other hand, when applied at higher dosages, AgNPs were internalized into the head and mitochondrial region of spermatozoa. We also found that AgNPs treatment could alter the normal mitochondrial architecture and increased the mtDNA copy number, thereby indicating that mitochondrial abnormalities may arise due to AgNPs exposure. This was consistent with previous results that found that AgNPs could disrupt the mitochondrial respiratory chain, thereby reducing the mitochondrial activity of sperm^39^.

Next, we performed the morphological analysis of spermatozoa and observed that AgNPs exposure significantly increased sperm abnormalities, including coiled tails, roll tail, and bent tails, in a dose-dependent manner. Besides, our results showed that AgNPs exposure decreased the percentage of live spermatozoa or sperm viability with increasing dose. Our findings are in agreement with several other reports that have shown that AgNPs uptake in animal models decreased the total sperm count and increased abnormal sperm shape^30^,^40^. Also, several *in vitro* studies have demonstrated the dose-dependent adverse effects of AgNPs on sperm viability^26^. In the present study, AgNPs exposure also increased the intracellular ROS formation in sperm and pre-treatment with the ROS inhibitor NAC for 30 min significantly decreased the AgNPs-induced alterations in sperm viability and mtDNA copy number. It has been reported that AgNPs-induced ROS are responsible for sperm abnormality and reduced sperm viability^30^,^39^. Excess ROS can induce sperm abnormality via peroxidative damage of the sperm plasma membrane^41^,^42^,^43^,^44^,^45^,^46^. Mangelsdorf *et al.*^40^ and Aziz *et al.*^47^ showed that ROS levels are positively correlated with the proportion of spermatozoa with amorphous heads, damaged acrosomes, midpiece defects, cytoplasmic droplets, and tail defects. However, in our experiments, we could not use Ag^+^ ions as a positive control, as it formed a precipitate in modified Whitten’s medium (formation of insoluble AgCl, Ag_3_PO_4_, etc). Therefore, AgNPs may be the main reason for the toxicity of spermatozoa. Collectively, these data suggest that AgNPs-induced reduction in sperm viability and abnormal sperm morphologies appear to be closely associated with increased ROS production and mitochondrial damage.

A unique function of spermatozoa is its interaction with the oocyte for fertilization. This interaction requires several essential physiological changes in order to be competent for oocyte fertilization; this process is termed as capacitation^48^. Sperm capacitation can be assayed by the sperm’s ability to undergo the acrosome reaction and the detection of a membrane cofactor protein, CD46, a reliable marker of acrosome-reacted spermatozoa^49^,^50^. Our results showed that AgNPs exposure significantly decreased the sperm acrosome reaction (CD46-negative) in a dose-dependent manner, thereby proving its negative impact on fertility. This result is consistent with previous reports where the authors have shown that the acrosome integrity of spermatozoa from AgNPs-treated animals was impaired^34^,^39^. This is possibly due to the pro-oxidant effects of AgNPs, which increased the rate of ROS generation^42^,^51^ and affected the acrosome reaction^34^,^39^.

In our present study, we found that AgNPs treatment had a negative effect on oocyte fertilization and subsequent embryonic development, especially late development, to expanded blastocyst and hatching blastocyst stages with increasing dose of AgNPs. This detrimental effect of AgNPs on fertilization is probably due to abnormal sperm morphology, impaired acrosome integrity and reduced sperm viability^48^,^52^,^53^,^54^,^55^,^56^. Therefore, our results suggest that AgNPs might reduce the fertilization capacity of spermatozoa.

During normal embryogenesis, at the blastocyst stage, the TE cells from the trophoblast develop a sphere made up of epithelial cells, which surrounds the ICM and blastocoel. These TE cells are required for development of the embryonic portion of the placenta and mammalian conceptus^57^,^58^. In addition, the ICM are pluripotent cells, which give rise to the embryonic tissue that comprises the ectoderm, endoderm, and mesoderm^59^. Li *et al.*^37^ suggested that AgNPs and Ag^+^ were potential cytotoxic agents for embryos that exert their effects through induction of cell apoptosis in ICM and TE cells of blastocysts, leading to decreased embryonic development and viability. Our results support this report: we showed that AgNPs-treated spermatozoa significantly inhibited ICM and TE cell proliferation in blastocysts and downregulated ICM- and TE-specific gene expression that plays a crucial role in ICM and TE cell formation.

Morphological abnormality in sperm is strongly correlated with fertilization failures *in vitro*^60^. With the help of ICSI, which allows oocyte fertilization irrespective of spermatozoon morphology and viability characteristics, we sought to determine the success of ICSI using dead spermatozoa with abnormal morphology due to AgNPs exposure. We observed that dead spermatozoa with normal morphology, coiled, or bent tails obtained from AgNPs treatment could reduce the fertilization rate. Our results suggested that integrity of the genomic DNA in sperm cells with abnormal morphology was not damaged by AgNPs treatment.

Moreover, dead spermatozoa with normal morphology or coiled or bent tails after exposure to AgNPs significantly inhibited ICM and TE cell proliferation in blastocysts, and also down-regulated ICM- and TE-associated genes. From our results, most of the blastocysts that were fertilized via AgNPs-treated spermatozoa resulted in delayed development, as well as decreased ICM and TE cell numbers. Bos-Mikich et al.^61^ and Lundin *et al.*^62^ suggested that a large number of early-cleaving embryos become good quality embryos and significantly facilitate high pregnancy, implantation, and birth rates. Therefore, we expect that AgNPs may affect embryo implantation. As shown in a previous report, the critical concentration of AgNPs (5–46 nM) that resulted in embryonic abnormalities and death was found to be 1.9 nM^63^. Besides, AgNPs (50 μM) induced a high resorption rate of post-implantation embryos and a decrease in fetal weight^37^. Similarly, Philbrook *et al.*^64^ showed that administration of AgNPs to pregnant CD-1 mice resulted in reduced fetus viability.

In conclusion, AgNPs are a potential cytotoxic agent for sperm cells and exert adverse effects, possibly through the induction of oxidative stress. Furthermore, AgNPs-treated sperm reduce the IVF success rate, delay subsequent blastocyst formation, and downregulate gene expression responsible for embryonic development. Our present *in vitro* study will offer further mechanistic insights into the effects of AgNPs on mammalian sperm physiology, such as the underlying mechanism or maximum size limit for AgNPs internalization into sperm cells.

## Methods

### Animals

Male and female BDF1 mice (8–12 weeks old) were housed in wire cages at 22 °C ± 1 °C with 70% humidity under a 12/12 h light–dark cycle. Mice had access to food and water *ad libitum*. These BDF1 mice are cross between female C57BL/6 and male DBA/2 mice. They are small in size, have short life cycle and easy to take care. Importantly, they are reposed on hormone injection during super ovulation and produce large number of oocytes. That is why we used BDF1 mice in our present study. This study was carried out in strict accordance with the recommendations in the Guide for the Care and Use of the Konkuk University Animal Care and Experimentation Community. The protocol was approved by the Committee on the Ethics of Animal Experiments of the Konkuk University (IACUC approval number: KU11035). All surgeries were performed under sodium pentobarbital anesthesia, and all efforts were made to minimize suffering.

### Materials

AgNPs were obtained from Nano High Tech (South Korea) as a clear colloidal aqueous suspension with a concentration of 1000 PPM (or mg/L). AgNPs were dissolved in a modified Whitten’s medium (118.5 mM NaCl, 4.7 mM KCl, 1.18 mM KH_2_PO_4_, 2.54 mM CaCl_2_, 1.18 mM MgSO_4_, 24.9 mM NaHCO_3_, 5.56 mM glucose, and 3 mg/mL BSA) or non-capacitation (NCP, without BSA) and prepared at a final concentration of 0.1, 1, 10, or 50 μg/mL just before treatment. NaCl, KCl, KH_2_PO_4_, CaCl_2_, MgSO_4_, NaHCO_3_, Glucose, and BSA were purchased from Sigma-Aldrich (St. Louis, MO, USA).

### AgNPs characterization

AgNPs were primarily characterized by UV-visible spectroscopy. Ultraviolet-visible (UV-vis) spectra were obtained using WPA (Mechasys, South Korea). The particle size distribution analysis was carried out using DLS measured by Zetasizer Nano ZS90 (Malvern Instruments, Ltd., UK). XRD patterns were obtained using an X-ray diffractometer (Bruker D8 DISCOVER, Bruker AXS GmBH, Karlsruhe, Germany). A transmission electron microscope (TEM; JEM-1200EX) was used to determine the size and shape of AgNPs.

### Spermatozoa preparation and AgNPs treatment

Spermatozoa were derived from the ampulla of the vas deferens of male BDF1 mice (8–12 weeks old). The ampulla was dissected with scissors and then squeezed by forceps compression. The fluid was rapidly collected and gently suspended in 200 μL of a modified Whitten’s medium containing 3 mg/mL BSA. After that, the suspension was incubated at 37 °C under humidified atmosphere of 5% CO_2_ in air for 30 min. The spermatozoa solution was placed in the bottom of a 5-mL snap tube containing 1 mL of modified Whitten’s medium and incubated at 37 °C under humidified atmosphere of 5% CO_2_ in air for 30 min to allow live spermatozoa to swim up. The spermatozoa suspension (800 μL) was collected from the top of the tube and centrifuged at 270 g for 5 min. The pellets were resuspended in modified Whitten’s medium, adjusting the concentration of spermatozoa before incubation with different concentration of AgNPs. Ten microliters of the spermatozoa suspension (2 × 10^4 ^cells/μL) were dropped into pre-warmed modified Whitten’s medium containing 0.1, 1, 10, and 50 μg/mL AgNPs and incubated at 37 °C under humidified atmosphere of 5% CO_2_ in air for 3 h. In the *N*-acetyl-l-cysteine (NAC) pre-treated group, the sperm were first treated with 5 mM NAC for 30 min and followed by AgNPs-treatment with different doses for additional 3 h.

### Sperm morphology analysis

The samples were 10-fold diluted in a buffered formal saline solution (34.72 mM Na_2_HPO_4_, 18.68 mM KH_2_PO_4_, 92.4 mM NaCl, 4% (v/v) formaldehyde) and examined by wet preparations and observed by phase contrast microscopy^39^. A minimum of 100 spermatozoa were used for each experiment. Three independent experiments were done to evaluate the morphology. Spermatozoa were classified into 5 categories: normal morphology, detached head, coiled tail, roll tail, and bent tails.

### ROS assay

After treatment sperm cells were incubated with 10 μM 2′,7′-dichlorodihydrofluorescein diacetate (DCFH-DA; Sigma-Aldrich, D6883) at 37 °C for 30 min in modified Whitten’s medium and washed with PBS. DCFH-DA-positive cell populations were identified using a FACSCalibur cell analyzer (Becton Dickinson, Franklin Lakes, NJ, USA).

### Dead and live analysis of spermatozoa

The number of dead and live spermatozoa was assessed using a LIVE/DEAD Sperm Viability Kit (Molecular Probes, Oregon). Briefly, samples were incubated with 100 nM of SYBR 14 dye at 37 °C under humidified atmosphere of 5% CO_2_ in air for 10 min in the dark, and then incubated with 12 μM of PI for 5 min. After that, samples were analyzed by fluorescence microscopy and flow cytometry. For fluorescence microscopy, ten microliters of the stained spermatozoa were placed on a microscope slide coated with 0.1% poly-d-lysine (Sigma-Aldrich) and covered with a cover glass. The spermatozoa showing a bright green fluorescence were considered to be alive, while spermatozoa with red fluorescence were rated as dead. The percentage of dead or live spermatozoa was calculated using the following formula: (number of dead or live spermatozoa × 100)/total sperm count. Moreover, dead or live spermatozoa were confirmed using flow cytometry (FACS). The green fluorescent events that passed through a 525-nm band-pass filter were collected as the log of green fluorescence 1 (FL1). The red fluorescence parameter was operated as fluorescence 2 (FL2), through 575-nm band-pass filters. For each sample, a total of 10,000 spermatozoa were analyzed for the log of their fluorescence. The generated data were then analyzed for the relative fluorescence of the LFL1 and LFL2 using the Coulter Histogram Analysis program.

### Sperm acrosome reaction analysis

Sperm cells were washed twice by centrifuging at 270 g for 5 min. The samples were fixed with 2% paraformaldehyde at room temperature for 10 min and again washed twice. After that, the samples were incubated overnight with PBS containing 1% BSA to block nonspecific sites, followed by washing with PBS. The pellet was suspended in PBS and divided into two aliquots. To 1 aliquot, 1:50 diluted mouse anti-CD46 antibody was added and incubated at 4 °C for 30 min, followed by washing with PBS twice. Then, the pellet was resuspended with the secondary antibody (1:200) and incubated in the dark for 30 min. After washing, spermatozoa were placed on a microscope slide coated with 0.1% poly-d-lysine, covered with a cover glass, and observed by fluorescence microscopy. The other aliquoted samples were then analyzed by FACS as described previously. The generated data were then analyzed for the relative fluorescence of the LFL1 and cell number using the Coulter Histogram Analysis program.

### Scanning electron microscopy (SEM) and transmission electron microscopy (TEM)

For SEM analysis, AgNPs-treated sperm were fixed in 2.5% paraformaldehyde-glutaraldehyde (4 °C, phosphate buffer, pH 7.4) for 2 h and then post-fixed in 1% OsO_4_ (4 °C, phosphate buffer) for 2 h. Samples were rinsed with PBS to remove the fixative. Fixed sperm cells were dehydrated in EtOH (70% > 80% > 90% > 95% > 100%). Sperm cells were then dried in a critical point dryer (Hitachi SCP-II), coated with gold using an IB-5 ion coater (Eiko), and observed under SEM (S-4700, Hitachi, Japan, 15 kV). Backscattered electron images in the SEM display compositional contrast that results from different atomic number elements and their distribution. Energy Dispersive Spectroscopy (EDS) allows one to identify what those particular elements are and their relative proportions (Atomic % for example).

For TEM analysis, sperm cells were fixed, washed, and then dehydrated in EtOH as before. After that, the sperm cells were embedded in Epon-Araldite mix solution and blocked at 60 °C in a vacuum drying oven (Yamoto, DPF-31) for 36 h. First, semi-thin slides were made using an ultramicrotome (LKB-2088) and stained with 1% toluidine blue (1% borax) on a 60 °C hot plate for 2 min. After that, to observe the sperm cell micro-structures, we made ultra-thin slices and stained with uranyl acetate and lead citrate. Examination of sections was performed with a transmission electron microscope operated at 100 kV.

### ***In vitro*** fertilization (IVF)

Female BDF1 mice (6–8 weeks) were superovulated with the help of intraperitoneal injection of pregnant mare’s serum gonadotropin, PMSG (10 units). After 48 h, they were injected by human chorionic gonadotropin, hCG (10 units). Metaphase II (MII) oocytes with cumulus cells were collected from the oviductal ampulla 12–14 h after hCG injection, and then placed in a 50-μL drop of modified Whitten’s medium covered with mineral oil. MII oocytes were incubated in modified Whitten’s medium at 37 °C under humidified atmosphere of 5% CO_2_ in air at least 1 h before co-incubation with treated sperm. Spermatozoa derived from each AgNPs treatment were washed by centrifugation with 270 g for 5 min twice. Then, spermatozoa pellets were resuspended with 50 μL of modified Whitten’s medium and mixed with the above 50 μL drop of modified Whitten’s medium. The oocytes were counted and equally divided into groups. After that, the spermatozoa and oocytes were co-incubated with modified Whitten’s medium at 37 °C under a humidified atmosphere of 5% CO_2_ in air for 6 h. Finally, zygotes were washed and cultured in KSOM medium at 37 °C under a humidified atmosphere of 5% CO_2_ in air for 96 h.

### Intracytoplasmic sperm injection (ICSI)

To generate spermatozoa that were completely dead, they were treated with 50 μg/mL AgNPs for 6 h in high dose. Spermatozoa were then suspended in 50 μL of 7% PVP medium in a 90-mm culture dish covered with oil. A piezo micromanipulator (PMAS-CT150; Prime Tech, Ibaraki, Japan) was connected with an inverted microscope (IX71; Olympus, Tokyo, Japan). The blunt tip of the ICSI-piezo pipette with a 7–9-μm inner diameter was used to draw spermatozoa. The oocytes were transferred to an ICSI dish with a CZB-H medium drop. The holding pipette was connected with the micromanipulator (Narishige, Tokyo, Japan) used to hold the oocyte. The oocytes are oriented with 1^st^ polar body at 6 or 12 O’clock position using the blunt tip of the ICSI-piezo pipette. The spermatozoa heads were sucked into the ICSI-piezo pipette, and then the blunt-end of the ICSI-piezo pipette was used to cut the zona pellucida using an intensity and frequency of the piezo pulse at about 2–3. The blunt-end of the ICSI-piezo pipette was pushed through the zona pellucida. The plasma membrane was penetrated by switching on the piezo set up and applying one piezo pulse with an intensity and frequency equal to 1. Only 1 spermatozoon was injected into the cytoplasm, and then, the ICSI-piezo pipette was gently pulled from the oocyte. This procedure was repeated until all spermatozoa heads in the pipette were injected. The injected oocytes were retained in the ICSI dish for 10 min at room temperature and then transferred to KSOM culture medium for culturing at 37 °C under humidified atmosphere of 5% CO2 in air for 96 h.

### Inner cell mass (ICM) and trophectoderm (TE) cell analysis

At 96 h after *in vitro* culture (IVC), the blastocysts were collected and washed twice in PBS containing 1% BSA. The blastocysts were then fixed in 4% paraformaldehyde for 40 min at room temperature. After that, the blastocysts were washed twice in 1% BSA in PBS. The embryos were incubated in 1% BSA with 0.1% Triton-X 100 in PBS overnight at 4 °C. The blastocysts were washed twice in 1% BSA in PBS (15 min each) and incubated with rabbit anti-OCT4 and mouse anti-CDX2 (1:50) (1:100) for 1 h at room temperature. After incubation in primary antibody, samples were washed three times with 1% BSA in PBS (15 min each). Then, samples were incubated with secondary antibody (1:200) in the dark for 1 h at room temperature. After incubation, samples were washed thrice, mounted on a slide, and observed by fluorescence microscopy. The differential labeling of OCT4 and CDX2 in blastocyst stage embryos was used to determine the number of ICM and TE cells, respectively.

### Gene expression analysis

Fifteen blastocysts derived from each treatment were collected at 96 h after fertilization. The blastocysts were washed with diethylpyrocarbonate (DEPC) water. After that, blastocysts were collected into 5 μL of DEPC water, and kept at −80 °C. For total mRNA extraction, the mRNA from collected blastocysts was exacted by freezing in LN_2_ and thawing in water (37 °C) 5 times. cDNA was synthesized by a Reverse Transcription Kit (Roche) in a final volume of 20 μL following manufacturer's instructions. The quantification of all gene transcripts (*sex determining region Y-box 2;SOX2, POU class 5 homeobox1;POU5F1, Kruppel-like factor 4;KLF4, Eomesodermin;EOMES,* caudal type homeobox 2*; CDX2,* and *keratin 8; KRT8*) was carried out in 3 replicates by real-time RT-qPCR on a Lightcycler apparatus using Lightcycler^®^FastStart DNA Master SYBR Green I via an ABI Applied Biosystems machine. The primer sequences for each gene are shown in Table S1. The relative gene expression was quantified and analyzed by the 2-ddCt method. In all experiments, *GAPDH* mRNA was used as an internal standard.

### Copy number analysis

For quantification of mitochondrial DNA (mtDNA), essentially the same protocol was used as for qPCR, with 20 ng total DNA used as template, and normalization of cytochrome b *(CYTB)* (forward primer, ATTGACCTACCTGCCCCATC; reverse primer, CTCGTCCGACATGAAGGAAT) amplification level against the nuclear β-actin (forward primer, TCGCCATGGATGACGATA; reverse primer, CACGATGGAGGGGAATACAG) gene.

### Statistical analysis

All the experiments were performed in triplicate, and statistical analyses were performed. Figures show representative experiments. For statistical analysis, one-way analysis of variance (ANOVA) was performed to determine whether there were differences within the groups (P < 0.01), and Dunnett's *t*-test was performed to determine the significance of difference between the treatment and control group. Statistical tests were performed using Stat View version 5.0 (SAS institute, Cary, NC, USA).

## Additional Information

**How to cite this article**: Yoisungnern, T. *et al.* Internalization of silver nanoparticles into mouse spermatozoa results in poor fertilization and compromised embryo development. *Sci. Rep.* **5**, 11170; doi: 10.1038/srep11170 (2015).

## Supplementary Material

Supplementary Information
