# Supplementary material for: Internalization of silver nanoparticles into mouse spermatozoa results in poor fertilization and compromised embryo development
Source: Sci Rep. 2015 Jun 8;5:11170. doi: 10.1038/srep11170 (PMC4459204; doi:10.1038/srep11170)
Supplement: Supplementary Information [file srep11170-s1.pdf]

Internalization of silver nanoparticles into mouse spermatozoa results in poorfertilization and compromised embryo development

Ton Yoisungnern[1, 2†], Yun-Jung Choi[1*], Jae Woong Han[1], Min-Hee Kang[1], Joydeep Das[1], Sangiliyandi Gurunathan[1], Deug-Nam Kwon[1], Ssang-Goo Cho[1], Chankyu Park[1], Won Kyung Chang[1], Byung-Soo Chang[3], Rangsun Parnpai[2], and Jin-Hoi Kim[1*]

[1]*Department of Animal Biotechnology, College of Animal Bioscience and Biotechnology/Animal Resources Research Center, Konkuk University, Seoul 143-701, South Korea*

[2]*Embryo Technology and Stem Cell Research Center and School of Biotechnology, Suranaree University of Technology, Nakhon Ratchasima 30000, Thailand*

[3]*Department of Cosmetology, Hanseo University, Seosan, Chungnam 356-706, Korea*

Table S1. Developmental competence of mouse oocytes fertilized *in vitro* after culture 96 h after insemination.

Treatments	No. of IVF	No. of unfertilized (%, ±SE)	No. of embryos development to (%, ±SE)							
			2C	4C	8C	Mo	Early BL	BL	Hatching BL	Total BL
0 µg/ml AgNPs	135	15 (11.1±4.8)[c]	15 (11.1±4.0)	0 (0.0±0.0)	0 (0.0±0.0)	3 (2.2±2.1)	11 (8.1±5.4)	42 (31.1±2.8)[a]	49 (36.3±13.8)[a]	102 (75.5±10.6)[a]
0.1 µg/ml AgNPs	135	32 (23.7±8.5)[c]	26(19.3±8.0)	0 (0.0±0.0)	0 (0.0±0.0)	3 (2.2±2.0)	7(5.2±3.1)	36 (26.7±6.2)[ab]	31 (23.0±14.9)[ab]	74 (54.8±12.7)[ab]
1 µg/ml AgNPs	135	60 (44.4±10.2)[b]	23 (17.0±11.5)	0 (0.0±0.0)	2 (1.5±1.5)	4 (3.0±2.5)	7(5.2±1.3)	24 (17.8±2.4)[b]	17 (12.6±10.9)[ab]	48 (35.6±13.1)[bc]
10 µg/ml AgNPs	135	88 (65.2±4.2)[a]	25 (18.5±1.9)	0 (0.0±0.0)	2 (1.5±1.5)	1 (0.7±0.6)	8 (5.9±0.9)	5 (3.7±1.9)[c]	6 (4.4±4.4)[b]	19 (14.1±5.1)[c]
50 µg/ml AgNPs	135	104 (77.0±4.5)[a]	17 (12.6±4.2)	1 (0.7±1.2)	3 (2.2±1.6)	1 (0.7±0.4)	7 (5.2±1.3)	2 (1.5±1.3)[c]	0 (0.0±0.0)[b]	9 (6.7±2.2)[c]

[a,b,c] Different superscripts denote significant differences (ANOVA:Duncan's Multiple Range Test, P<0.05).

Mo and BL indicate molular and blastocyst, respectively.

Table S2. Developmental competence of mouse embryos derived from ICSI with dead AgNP-exposed sperm with different morphology after culture for 96 h.

Treatment	No. ICSI	No. unfertilized (%, ±SE)	No. embryos development to (%, ±SE)							
			2C	4C	8C	Mo	Early BL	BL	Hatching BL	Total BL
Live sperm	65	6 (9.2±1.0)[b]	10 (15.4±3.2)	6 (9.2±1.0)	0 (0.0±0.0)	7 (10.8±6.5)	9 (13.8±3.3)	16 (24.6±6.2)	11 (17.0±5.5)[a]	36 (55.4±5.3)[a]
Dead Normal morphology*	65	9 (13.8±2.0)[a]	14 (21.5±3.7)	9 (13.8±4.4)	0 (0.0±0.0)	11 (16.9±4.4)	6(9.2±4.2)	13 (20.0±6.4)	2 (3.1±2.9)[b]	21 (32.3±4.3)[b]
Dead detached head*	65	9 (13.8±1.7)[a]	13(20.0±5.5)	9 (13.8±2.5)	1(1.5±2.0)	9(13.8±6.3)	9(13.8±4.3)	12 (18.5±6.2)	3 (4.6±5.8)[b]	24 (36.9±7.9)[b]
Dead coil tail*	65	12 (18.5±3.5)[a]	14 (21.5±2.5)	9 (13.8±3.1)	2 (3.1±2.1)	9 (13.8±4.7)	6 (9.2±3.7)	13 (20.0±4.9)	0 (0.0±0.0)[b]	19 (29.2±2.9)[b]

*Dead sperm derived from AgNP exposed and different morphology. [a,b]Different superscripts denote significant differences (ANOVA:Duncan's Multiple Range Test, P<0.05). Mo and BL indicate molular and blastocyst, respectively.

Table S3. Primers sets used for real-time qRT-PCR.

Primer name	Primer sequence	Annealing	Size (bp)
GAPDH	F: AGGTCGGTGTGAACGGATTTG<br>R: TGTAGACCATGTAGTTGAGGTCA	57℃	123
Pou5f1	F:CTCCCTACAGCAGATCACTCACA<br>R : AACCATACTCGAACCACATCCT	63℃	220
Sox2	F: CACAACTCGGAGATCAGCAA<br>R: CTCCGGGAAGCGTGTACTTA	60℃	190
Klf4	F: CTGAACAGCAGGGACTGTCA<br>R: GTGTGGGTGGCTGTTCTTTT	58℃	218
Cdx2	F : AGACAAATACCGGGTGGTGTA<br>R : CCAGCTCACTTTTCCTCCTGA	60℃	153
Eomes	F:GAGCTTCAACATAAACGGACTCAA<br>R : CGGCCAGAACCACTTCCA	60℃	210
Krt8	F : ATCGAGATCACCACCTACCG<br>R : TGAAGCCAGGGCTAGTGAGT	63℃	151

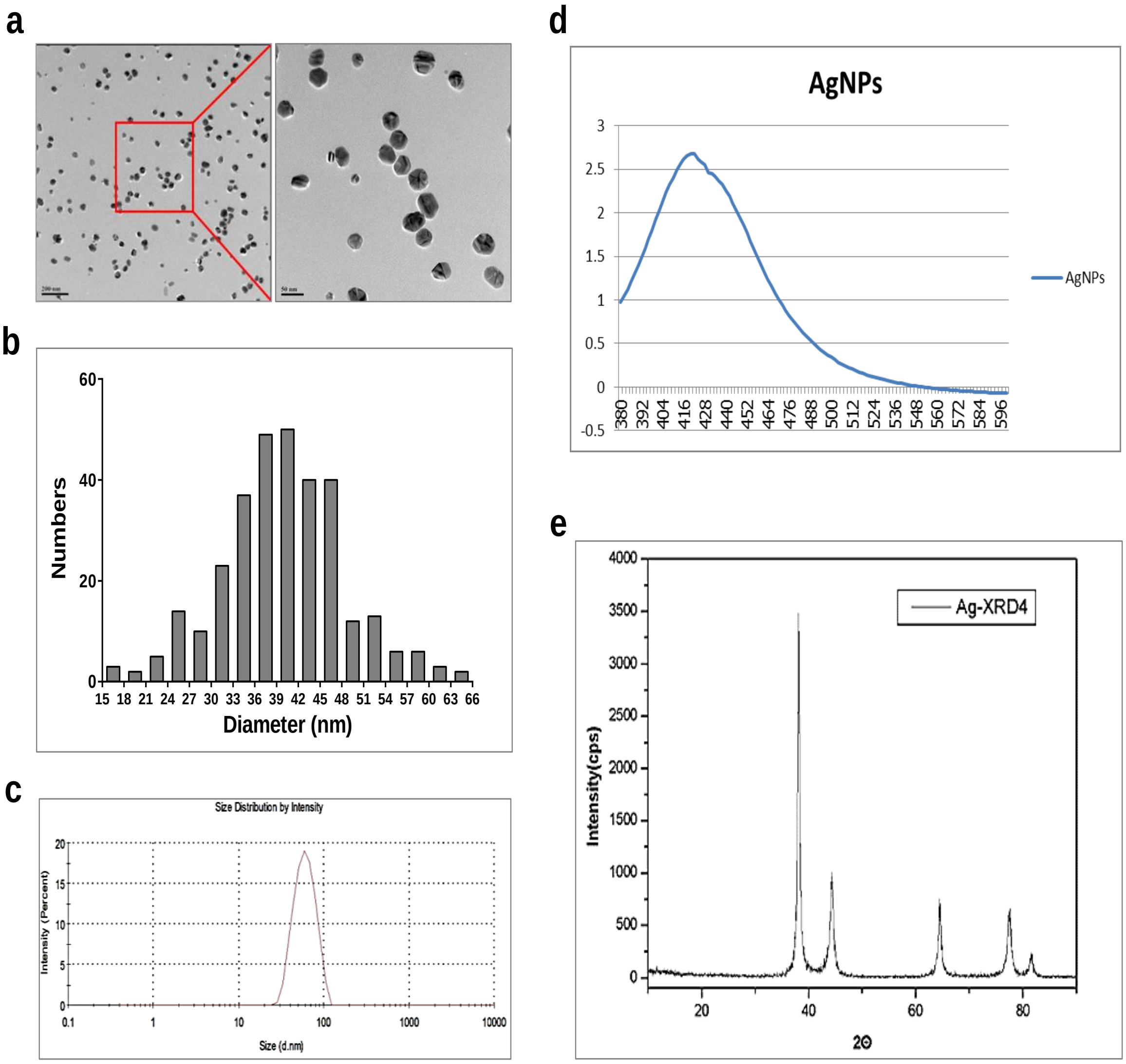

Figure S1. Characterization of AgNPs. (a) Size and morphology of AgNPs as measured by TEM. (b) Several fields were photographed and used to determine the diameter of nanoparticles. The average range of observed diameters was 40 nm. (c) Size distribution analysis by DLS. (d) The absorption spectrum of AgNPs. The absorption spectrum of AgNPs exhibited a strong broad peak at 420 nm, and observation of such a band is assigned to surface plasmon resonance of the particles. The samples were visualized in UV-vis spectra. (e) XRD pattern of AgNPs.

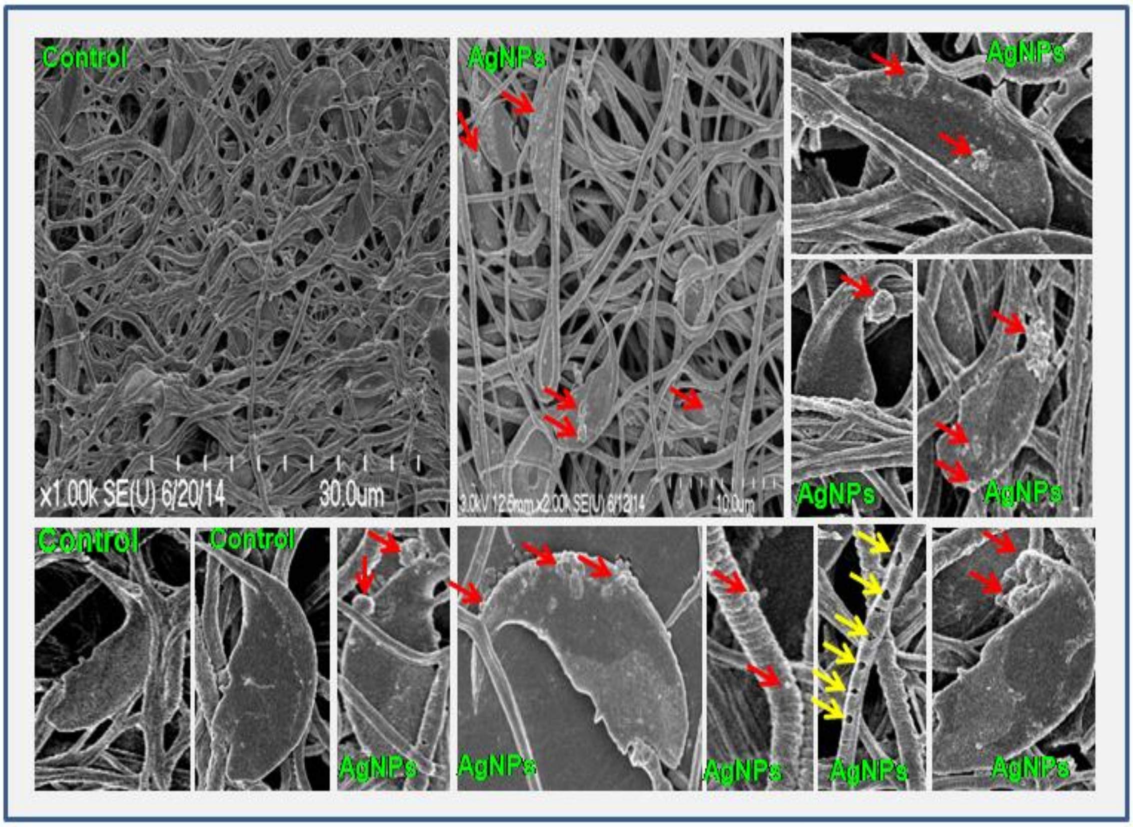

Figure S2. Localization of AgNPs in sperm cells by SEM. Sperm cells were co-cultured with or without AgNPs (1, 10 and 50g/mL) for 3 h. AgNPs were detected in the AgNP-treated group (indicated by arrows) but not in the control. Red and yellow arrow indicate the presence of AgNPs on sperm head and damaged tails, respectively.

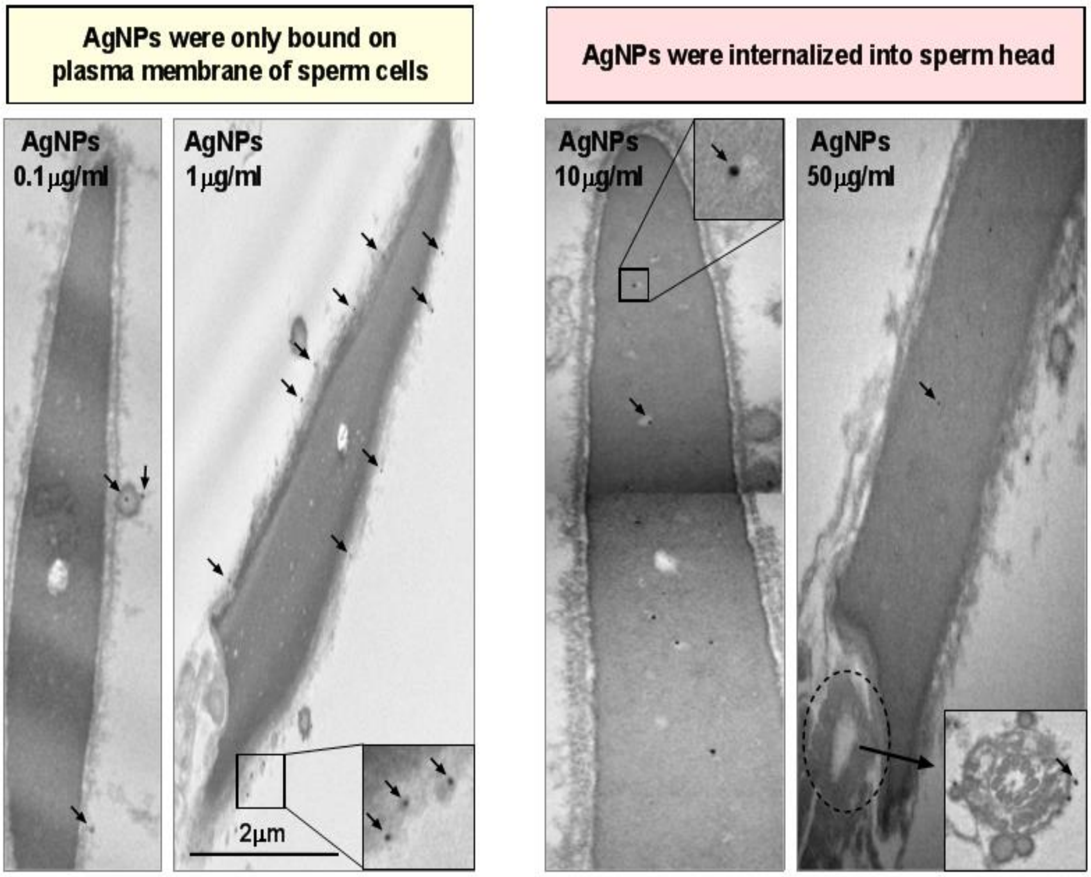

Figure S3. Localization of AgNPs in sperm cells by TEM. Sperm cells were co-cultured with AgNPs (0.1, 1, 10 and 50g/mL) for 3 h. Arrows indicate AgNPs inside sperms. At low dosages, AgNPs mainly localized on the sperm plasma membrane, whereas with increasing dosage, AgNPs were internalized into the head of the spermatozoa.

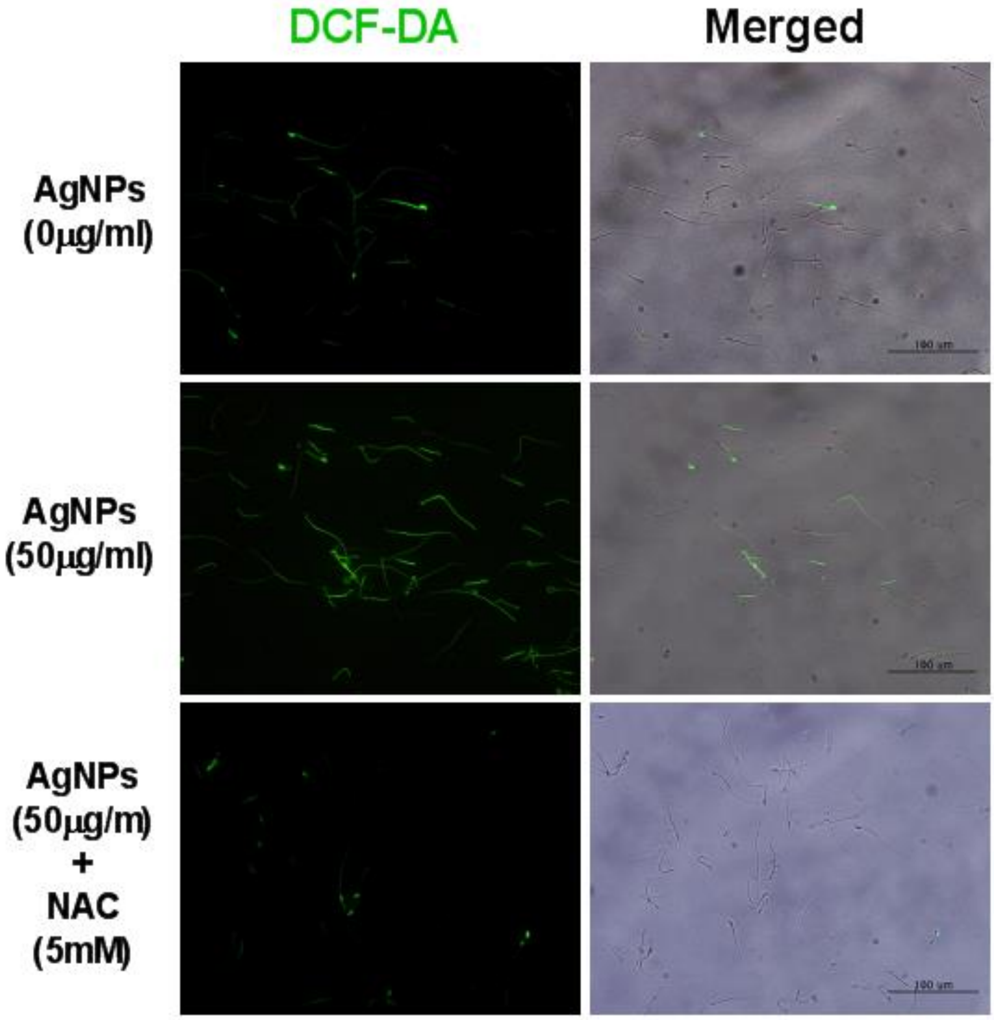

Figure S4. NAC treatment decreases AgNPs-mediated oxidative stress in sperm cells. Sperm cells were exposure to 50μg/mL of AgNPs for 3 h with or without pre-exposure to NAC for 30 min. DCFH-DA-FITC was measured using fluoromicroscopy analysis. Scale bar = 100 μm. Note, DCFH-DA-FITC positive signals in living sperm cells is mainly localized onto middle piece of sperm tail, whereas DCFH-DA-FITC positive signals in dead sperm cells are localized in whole sperm cells.

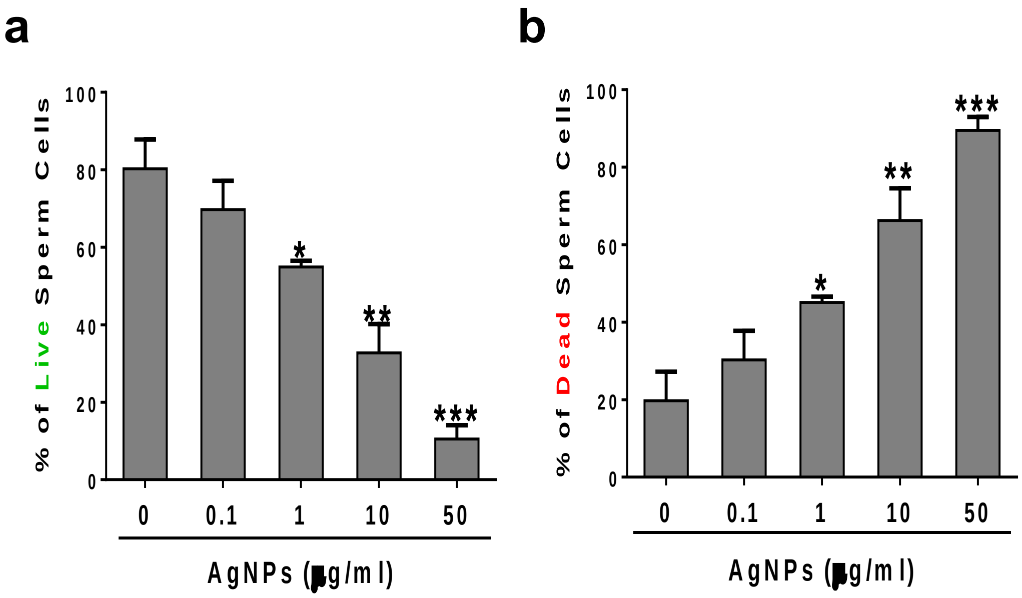

Figure S5. Analysis of live (a) and dead (b) sperm cells in non-capacitation medium after AgNP exposure. Sperm cells were cultured in non-capacitation medium with different concentration of AgNPs for 3h. Dead and live sperm cells were stained using the Dead/Live kit and counted by flow cytometry. $P^{*}<0.05$, $^{**}P< 0.01$, $^{***}P< 0.001$ versus the control group (Dunnett's t Tests).

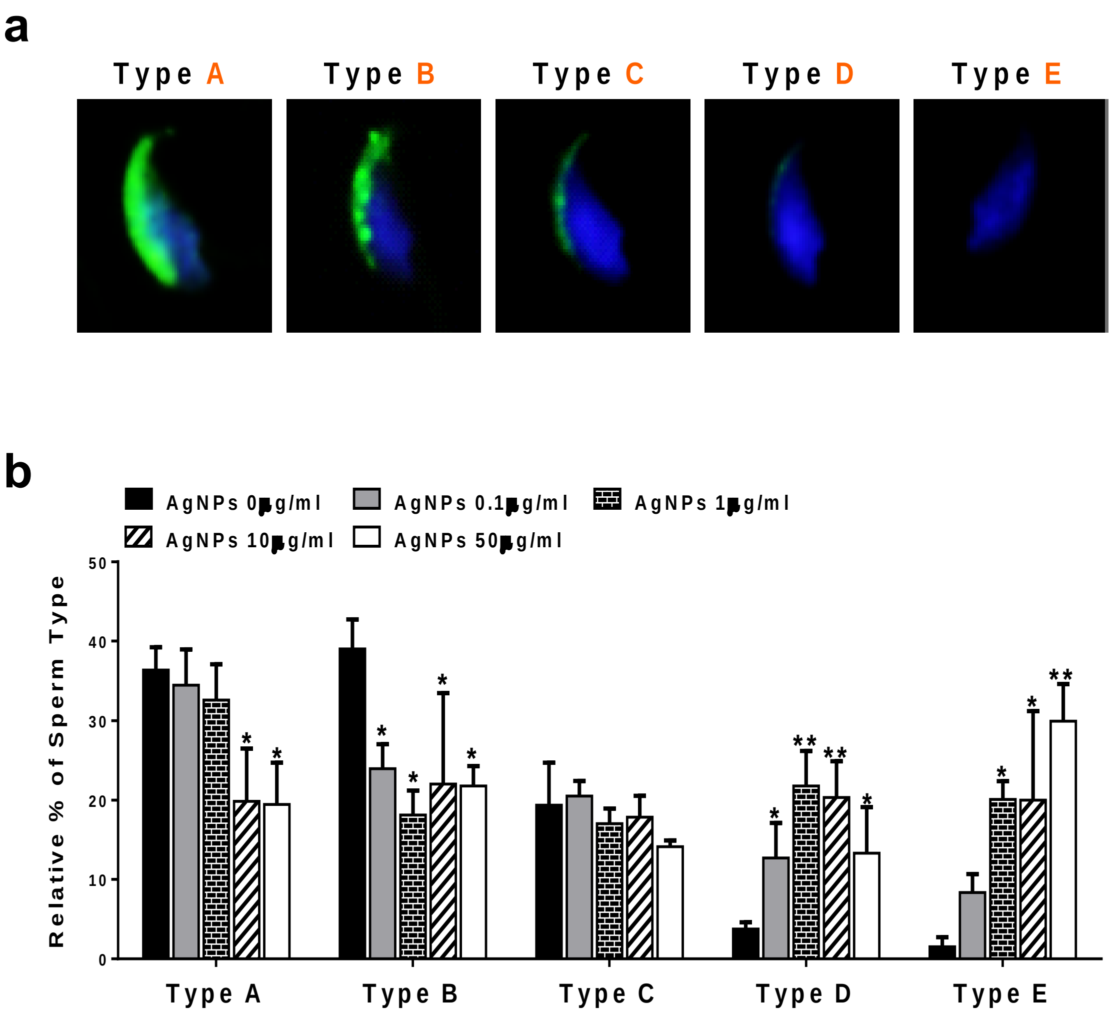

Figure S6. CD46 immunofluoresent staining intensity analysis in sperm cells cultured in non-capacitation medium after incubation with different dosage of AgNPs. (a) Representative immunofluoresent staining patterns using mouse anti-CD46 (green). Nuclei are counterstained with DAPI (blue). (b) Each immunofluoresent staining pattern was analyzed by using anti-CD46 antibody after treatment with different dosage of AgNPs. $^{*}P < 0.05$ and $^{**}P<0.01$ versus the control group (Dunnett's t Tests).

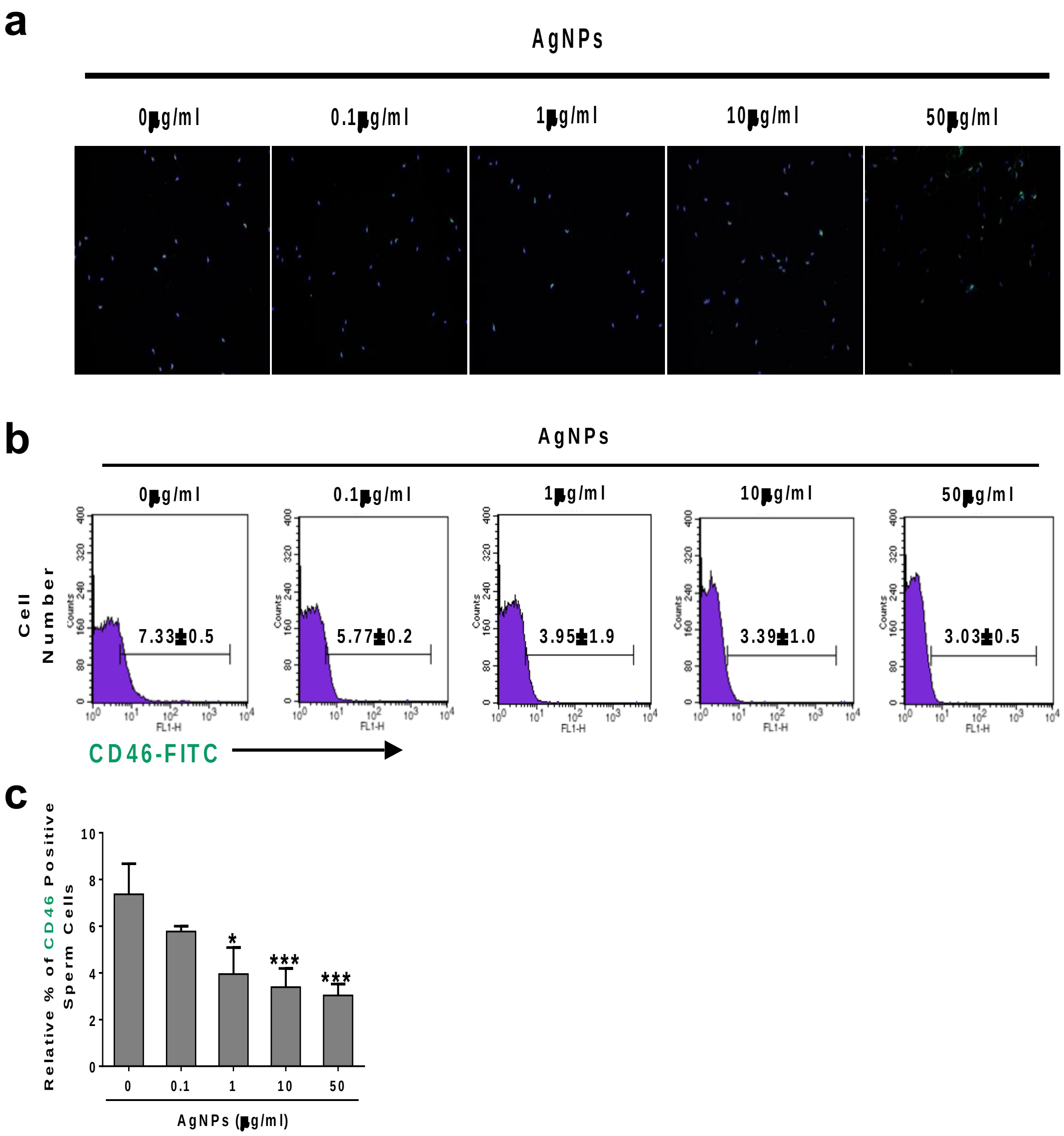

Figure S7. Acrosome reaction analysis in sperm cells cultured in non-capacitation medium using flow cytometry. Sperm cells were cultured in non-capacitation medium with different concentration of AgNPs for 3h. (a) A representative immunofluoresent staining pattern obtained by using mouse anti-CD46 (green) antibody. Nuclei are counterstained with DAPI (blue). (b, c) Flow cytometry analysis of CD46-positive sperm populations. $^{*}P < 0.05$, $^{**}P<0.01$, $^{***}P< 0.001$ versus the control group (Dunnett's t Tests).

a

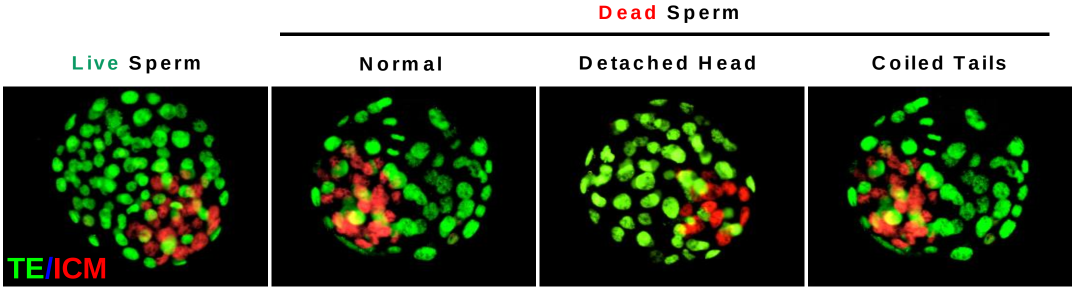

b

Treatment	No. of Blastocysts	No. of Total Cells (Mean±SE)	No. of ICM Cells (Mean±SE)	No. of TE Cells (Mean±SE)
Live	15	84.3±3.8^{a}	18.4±1.1^{a}	65.9±3.4^{a}
Dead Normal	11	57.2±3.2^{b}	12.3±1.0^{b}	44.6±3.0^{b}
Dead Detached Head	10	65.2±2.7^{b}	14.3±1.0^{b}	50.9±2.2^{b}
Dead Coiled Tails	10	55.0±3.3^{b}	12.0±1.2^{b}	43.0±2.6^{b}

c

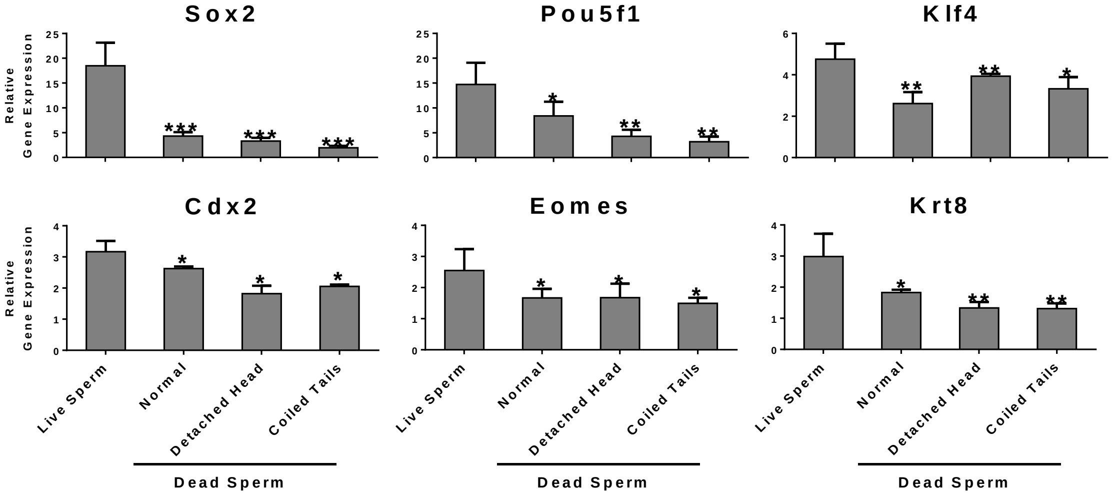

Figure S8. Qualitative analysis of blastocyst stage embryos developed in vitro after ICSI with AgNPs-treated sperm cells with normal or abnormal morphology. a) A representative blastocyst stained differentially by Oct4 and Cdx2 antibodies 96 h after ICSI with dead normal or abnormal sperm. Oct4-positive cells (red) are putative inner cell mass (ICM), whereas Cdx2-positive cells (green) are putative trophectoderm. b) The number of total cells, ICM, and TE cells from the blastocyst stage embryos of (a). Different superscripts denote significant differences (ANOVA:

Dunnett's t Tests, $p < 0.01$). c) ICM- and TE-specific mRNA expression analysis in blastocysts developed after ICSI with AgNPs-treated normal or abnormal sperm cells. The expression levels of ICM and TE associated genes were analyzed by real time RT-qPCR. The experiments were performed in triplicate; data represent the mean of three independent experiments. Error bars represent standard error of the mean (SEM). $^{*}p < 0.05$, $^{**}p < 0.01$ versus the control group (Dunnett's t-tests).
